# Coexistence research requires more interdisciplinary communication

**DOI:** 10.1002/ece3.8914

**Published:** 2022-05-13

**Authors:** Hadas Hawlena

**Affiliations:** ^1^ Mitrani Department of Desert Ecology Jacob Blaustein Institutes for Desert Research Ben‐Gurion University of the Negev Midreshet Ben‐Gurion Israel

## Abstract

Coexistence theories develop rapidly at the ecology forefront, outpacing their experimental testing. I discuss the reasons for this gap, call on interdisciplinary researchers to construct a road map for coexistence research, and recommend the actions that should be implemented therein.

## Graphical abstract

Coexistence theories outpace their experimental testing. I argue that this imbalance results from a combination of interdisciplinary gaps, a disparity in research approaches, and the limited accessibility of the theory to experimentalists. I call on interdisciplinary researchers to construct a road map for coexistence research, recommend the actions that should be implemented therein, and illustrate how multiple mechanisms can be integrated under the same coexistence terms, thus constructing a research landscape that can create uniformity in their experimental designs.

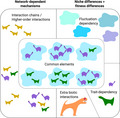

## 1 GAPS IN COEXISTENCE STUDIES

Coexistence is the long‐term co‐persistence of different species, with each species able to recover from low density (Chesson, 2000). Since the formulation of Gause's law, which states that species competing for the same limiting resource cannot indefinitely persist together (Gause, 1934), ecologists have searched for the mechanisms that underlie the high number of coexisting species in an apparently limited number of ecological niches in nature. Moreover, coexistence mechanisms provide the foundation for understanding higher level ecological patterns, such as productivity–diversity relationships, cascading effects, and food web fluctuations, with applied implications for the effects of global warming, species introductions, and biodiversity degradation on human health, wildlife composition, and ecosystem function. Thus, it is not surprising that interest in coexistence has remained constant. However, despite ecologists’ consensus that coexistence mechanisms must be deciphered, the theoretical development has outpaced its experimental testing and recent calls to improve the situation (Ellner et al., 2019) have not yet come to fruition. Based on my personal experience (see below) and a systematic literature review of experimental coexistence studies from 1967 to 2020 (H. Hawlena, M. Garrido, C. Cohen, S. Halle and S. Cohen, unpublished data), in my view, the shortage in well‐designed experimental studies is largely the combined result of interdisciplinary gaps, an imbalance in focusing the research on phenomenological, mechanistic, and system‐specific approaches, and the limited accessibility of the theory to experimentalists, as explained below.

## 2 INTERDISCIPLINARY GAPS

Experiments that test coexistence mechanisms are common in various fields, including microbial ecology, plant ecology, behavioral ecology, physiological ecology, evolutionary biology, community ecology, disease ecology, and spatial ecology. Interdisciplinary perspectives enable progress and are a cornerstone of science. However, in coexistence studies, due to the independent development of the mechanistic theories and the use of discipline‐specific terminologies and mechanistic formulations in the models, the potential of interdisciplinary approaches has been superseded by miscommunication and a tendency to “reinvent the wheel.” This miscommunication is represented in Table 1, which shows that the same mechanisms are often known by different discipline‐specific terms.

**TABLE 1:** A proposed hierarchical organization of various coexistence mechanisms under common terms

(A) Mechanisms allowing stabilizing niche differences to offset relative fitness differences					
Fluct. dep.?	Behavioral/biotic dependency		Specific mechanism	Trade‐off	Main drivers
Yes	Behavior‐independent		Storage effect^b^, ^c^	Tradeoff among species in resource use and associated mortality rates	Species traits allow gains made during favorable periods to be “stored” in the population for use during unfavorable periods (or environments)
	Behavior‐dependent mechanisms		Relative non‐linearities^b^, ^c^		Species have different nonlinear responses to competition, leading to fluctuations in the intensity of competition over time or space
			Spatial and temporal partitioning	The traits that make a species more competitive in one habitat (period) have drawbacks in another	Each species is more dominant in the habitat (periods) to which it is more adapted. Habitats differ in resource abundance, resource use, or enemy pressure **Habitat selection; habitat partitioning; conditional differentiation**
			Behavioral changes in fluctuating environments	The same behavior that is beneficial in one condition creates disadvantages in another	Species behaviors allow gains made during favorable periods (or environments) to be “stored” in the population for use during unfavorable periods **Trade‐offs in foraging costs among periods; foraging versus dormancy costs**
No	No extra biotic interactions; trait‐dependent mechanisms		Resource‐ratio^a^	Competitiveness for different limiting resources	Each species has a greater impact on the resource it finds most limiting **Resource partitioning; diet choice or separation; NDH**
			Competition‐tolerance^a^, ^b^	Competitiveness and resistance or tolerance	The superior competitor is less resistant or tolerant to abiotic conditions or disturbances **Competition‐resistance to disturbance; NDH**
			Exploitative‐interference competition	Interference and exploitative competitiveness	The inferior competitor is stronger in interference competition **Allelopathy and bacteriocin‐mediated competition**
			Competition‐colonization^b^	Competitiveness and colonization capabilities	Inferior competitors rapidly colonize available habitats before being outcompeted by superior competitors
	Extra biotic interactions	Generalist enemy or mutualist	Enemy‐ratio^a^	Each victim better tolerates a different enemy species	Each victim has a greater impact on the enemy that regulates it **Apparent mutualism; predator/enemy partitioning; NDH**
			Competition‐defense^b^	Competitiveness and resistance capabilities	The best competitor is more sensitive to exploitation by enemies **Food‐safety tradeoffs**
			FD‐exploitation	Population growth and exploitation risk	Enemies often disproportionately exploit their victims when abundant and disproportionately ignore them when rare **Switching; optimal foraging**
			Shared mutualists	Mutualism and interspecific competition	The mutualist that receives the highest benefit from one host provides a higher benefit to the species’ competitor, resulting in NFD dynamics **Plant‐soil feedbacks**
		Specialist enemy	Natural enemy partitioning	Population growth and exploitation risk	Specialized enemies increase their regulation with victim densities **Killing the winner hypothesis; plant‐soil feedbacks**
			Heteromyopia	None	Interspecific competition occurs over shorter distances than intraspecific competition, lowering the density of the abundant species

(B) Network‐dependent mechanisms		
Specific mechanism	Trade‐off	Main drivers
Interaction chains: e.g., intransitive interactions	The trait that helps a species to outcompete some competitors provides a disadvantage versus others	There is no single dominant competitor; thus, species outcompete some competitors while losing to others **Rock‐paper‐scissors games**
Higher‐order interactions	Competitiveness against multiple species	The presence of one/a few species weakens the interspecific interaction of other species combinations **Trait‐mediated indirect interactions; cascading effects**

Note

Numerous coexistence mechanisms have been proposed. Despite sharing similar goals, they reflect a broad spectrum of approaches, terminologies, scales, and schools of thought. This chart illustrates how the various mechanisms can be simplified (“main drivers”) and collapsed into a few general classes that are similar conceptually, to facilitate interdisciplinary communication. The nomenclature I chose comprises the underlying trade‐offs, and the associations with the broader classes are based on whether the mechanisms allow stabilizing niche differences to offset relative fitness differences (panel A) or whether they depend on the network structure of the interacting species and may promote species coexistence even without pairwise niche differences (panel B). The former class of mechanisms is further subdivided according to its dependence on environmental fluctuations, behaviour and extra biotic interactions. The bold terms in the right column are related to the specific discipline or study organisms, and the superscripted letters are also provided to associate terms with specific frameworks—jointly illustrating that the same mechanisms are sometimes known by different discipline‐specific terms. The list and division are not meant to be exhaustive but rather are attempts to show that the theory can bridge interdisciplinary gaps. I call on coexistence researchers to join hands and propose alternative divisions that will cross interdisciplinary barriers, research approaches, and model systems.

Abbreviations: FD‐exploitation, frequency‐dependent exploitation by generalist enemies; Fluct.dep., dependence on environmental fluctuations; NDH, niche dimension hypothesis; NFD, negative frequency‐dependent.

^a^ Represents the contemporary niche theories (Letten et al., 2017).

^b^ Can also be considered as an underlying mechanism for disturbance‐related coexistence models, such as the intermediate disturbance hypothesis (Shea et al., 2004).

^c^ Traditionally associated with the modern coexistence theory.

My perspective has emerged from personal experience. During my training as an experimental behavioral and community ecologist, coexistence studies focused on what we termed “the two obligatory coexistence requirements”: environmental heterogeneity and trade‐offs. My initial research area dealt mainly with resource, habitat, and temporal partitioning mechanisms, and to prove coexistence, my colleagues used habitat‐selection‐derived techniques (Morris, 2003). Later, when immersing myself in the field of evolutionary biology, I became familiar with the concept of interaction chains, learning that coexistence may actually occur in the absence of pairwise niche differences (Levine et al., 2017). However, to get to the core of the modern coexistence theory (MCT), I had to collaborate with a plant ecologist. MCT provides analytical ways to compare the importance of niche and fitness differentiating processes for species coexistence, focuses on the invasibility criterion for coexistence (the ability of each species to have a positive growth rate when rare in a resident community), and particularly considers the role played by environmental fluctuations (Chesson, 2000, 2018; Grainger et al., 2019). Surprisingly, as a seasoned disease ecologist, I realized that most of the above coexistence terms, concepts, requirements, and mechanisms are not embedded in the experimental studies of parasite/pathogen coexistence, which mostly refer to any co‐infecting parasites/pathogens (species that are observed together in the same host individual) as coexisting, without any experimental support (see an example in one of my own studies; Eidelman et al., 2019).

This variety of coexistence perspectives (Table 1) limits the theories’ accessibility to experimentalists, who are often only aware of the theories that fit their discipline‐specific terminologies, scales, and schools of thought. For example, Miller et al. (2005) reviewed the experimental evidence from 1980 to 2003 for Tilman's R^∗^ prediction of the resource‐ratio mechanism (Tilman, 1980) and found that only eight of the 13 valid studies support it. Unfortunately, Miller et al. overlooked 43 studies in the microbial and aquatic literature that allow rigorous tests of the concept because those works did not cite Tilman, 41 of which support the concept (Wilson et al., 2007). In other cases, the interdisciplinary gap may lead to multiple discoveries; see, for example, the bold terms in the column “main drivers” and the comments designated by the superscripted letters in Table 1. This is also illustrated by the presence of two parallel coexistence mechanisms, “natural enemy partitioning” and “killing the winner,” explored independently among plant community ecologists and microbial ecologists, respectively (Comita & Stump, 2020; Vage et al., 2018). The interdisciplinary gap may also result in a linkage between the target mechanism and the study system. This may explain why most studies of the resource‐ratio mechanism were tested in phytoplankton by microbial ecologists (Wilson et al., 2007), those of natural enemy partitioning were tested mostly in plants by plant ecologists (Comita et al., 2014; Comita & Stump, 2020), those of the food‐safety trade‐off were tested in vertebrates by behavioral ecologists (Kotler & Brown, 2020), and those of the “frequency‐dependent exploitation by generalist enemies” (hereafter, FD‐exploitation) were tested mainly in aquatic invertebrates by community ecologists (Sherratt & Harvey, 1993). Such mechanism‐system linkages limit the experimental ability to validate the theory more generally.

## 3 IMBALANCE IN RESEARCH APPROACHES

Coexistence research reflects a broad spectrum of approaches. These approaches can be roughly divided into three hierarchical classes. The highest is the phenomenological class, which deals with the universal conditions for coexistence. For example, in the hierarchical organization that I proposed, I grouped the mechanisms into two phenomenological classes (Table 1). The first is represented by the MCT, which hypothesizes that the invasibility criterion for coexistence occurs when competitors’ niche differences are greater than their fitness differences (panel A; Chesson, 2000; Mayfield & Levine, 2010). The second phenomenological class groups mechanisms that are based on the species’ network structure, and thus, mechanisms belonging to this class may promote coexistence even without pairwise niche differences (panel B; Levine et al., 2017). While these phenomenological classes constitute a common framework for a diverse range of study systems, mechanisms, and multiple topics, on their own, they provide no information about the exact mechanisms that determine the competitive outcome. The second, more mechanistic, class of approaches may fill this gap by exploring the factors that enable the above universal conditions for coexistence. For example, in my proposed hierarchical organization, I grouped the mechanisms that shape the niche and fitness differences between species according to their dependence on environmental fluctuations and extra biotic interactions (Table 1). The lowest class of approaches is more detailed and elaborated, often involving interactions between second‐class mechanisms and thus is not shown in Table 1. Its goal is to adjust the predictions to specific biological scenarios, e.g., the effect of enemy avoidance behavior on coexistence (Sommers & Chesson, 2019). This approach aims to untangle the exact conditions and combination of factors driving each system. Together, the three hierarchical approaches may complement our understanding of species occurrence patterns in nature. However, the literature trends suggest that in coexistence studies, the development of each approach has come at the expense of the others. The switch from the second and third hierarchical approaches, which prevailed from the 1970s to 2000s and the 2000s to 2010s, respectively, to the phenomenological approaches (2010 until the present), was accompanied by a drop in studies employing the former approaches. Thus, today, while the phenomenological knowledge of coexistence has rapidly accumulated (Godoy et al., 2014; Grainger et al., 2019; Levine et al., 2017; Mayfield & Levine, 2010; Saavedra et al., 2017; Spaak & De Laender, 2020), we still lack experiments that focus on specific mechanisms and that explore the specific selective factors underlying coexistence.

## 4 LIMITED ACCESSIBILITY OF THE THEORY TO EXPERIMENTALISTS

Converting the theory to testable predictions is challenging even without interdisciplinary gaps and multiple approaches. Thus, occasionally, the experiment is not suitably designed to address all criteria of the mechanism. For example, theory suggests that the storage effect mechanism promotes coexistence if two criteria are satisfied: (i) covariance between environment and competition must change between the resident (when the species is at its typical steady‐state abundance) and invader (when the species is at low densities) states and (ii) buffered population growth so that for each species, growth under favorable conditions compensates for the unfavorable conditions (Chesson, 2008). However, experimental studies exploring this mechanism are rarely designed to address these two criteria (Adondakis & Venable, 2004; Facelli et al., 2005; Li & Chesson, 2018; but see, Armitage & Jones, 2019; Hallett et al., 2019; Letten et al., 2018; Sears & Chesson, 2007). Such a theoretical‐empirical gap may reflect both the challenge of converting the mathematical insights into operational predictions and the tension between the high theoretical demands and the experimental constraints. This tension may partly explain the prevalence of experimental tests for the resource‐ratio mechanism (Wilson et al., 2007), which is relatively easy to test compared with the more limited studies testing the storage effect mechanism. In other studies, the theoretical assumptions are not justifiable. A typical example is the assumption of coexistence without conducting invasibility or alternative tests to differentiate coexistence from co‐occurrence (Siepielski & McPeek, 2010).

## 5 MOVING COEXISTENCE RESEARCH FORWARD

I propose moving coexistence research forward in a more mechanistic manner by restoring the coexistence experiments that go beyond purely phenomenological approaches to ones pursuing more specific mechanisms. This should proceed in parallel with continuous progress in the phenomenological approaches. My original intention was to bring the various coexistence mechanisms under the same umbrella. However, from the multiple, sometimes contrasting, comments that I have received on my initial ideas, I realized that this is not a “one‐person job.” I therefore call on coexistence theoreticians, modelers, and experimentalists from different disciplines to join hands, keep an open mind, and construct a road map for coexistence experiments that will cross interdisciplinary barriers, study approaches, and model systems. Below, I recommend the actions that should be implemented in future interdisciplinary discussions.

First, let's improve communication and merge the multiple discoveries by *integrating all mechanisms under the same coexistence terms*. In Table 1, I illustrate how various mechanisms can be simplified (column “main drivers”) and collapsed into a few general classes that are similar conceptually, to facilitate interdisciplinary communication. My division is based on the mechanism's dependence on the network structure (a phenomenological division), environmental fluctuations, species traits, and extra biotic interactions (mechanistic‐specific divisions), and the nomenclature relies on the underlying trade‐offs. However, this is just one option, and alternative divisions that cross interdisciplinary barriers can be similarly made (e.g., a division based on the spatial scale of the mechanism). I use this division here as a “proof of concept”—to advocate for using the theory to build interdisciplinary bridges in coexistence research.

Second, for each mechanism, let's *define the underlying assumptions and predictions in an operational way*. Miller et al. (2005) provided a good example that should be reproduced for all of the mechanisms introduced in Table 1.

**FIGURE 1:**
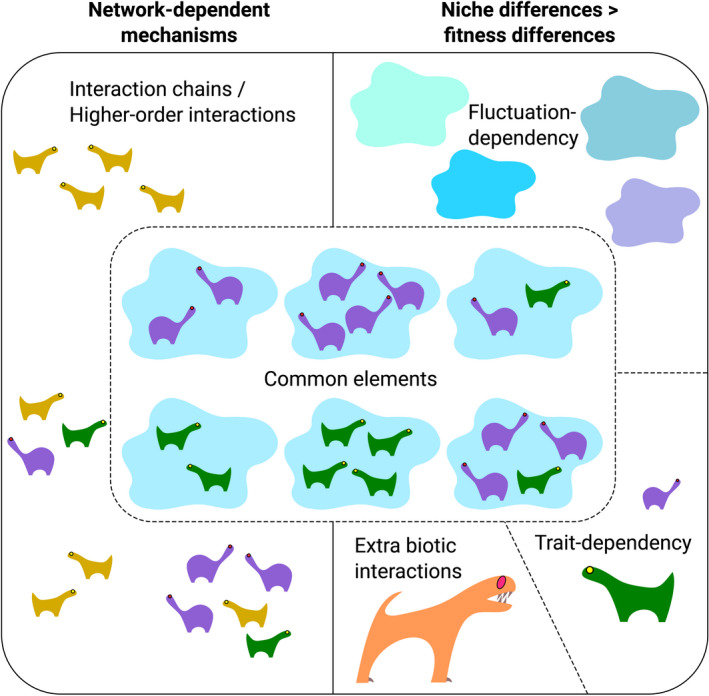
A proposed landscape of experimental designs that should be included in coexistence experiments. Experimental coexistence studies reflect a broad spectrum of designs, depending on the researcher's discipline, model organism, and tested mechanism. To facilitate interdisciplinary communication and uniform coexistence studies, I call on researchers to define a desirable experimental design for each coexistence mechanism, using common terms. The proposed landscape of experimental designs is based on the hierarchical organization that is presented in Table 1. Its center includes the density and species frequency components that should be included in any coexistence experiment, and the elements at the edges represent additional components that are required for specific mechanisms. The specific supplement depends on the mechanism's phenomenological class (left and right sides of the figure) and its reliance on environmental fluctuations (upper and lower parts of the figure's right side), species traits (right corner at the bottom), and extra biotic interactions (lower middle section). This landscape reflects the similarity in experimental designs, where mechanisms that are clustered in the same class (Table 1) or in adjacent classes that are divided by a dashed line can be simultaneously tested by the same experimental design. The cloud‐like shapes represent the organism's environment. The purple, green, and yellow organisms represent three competing species, whereas the orange organism is their enemy. All the organisms are intentionally not associated with a specific taxon or sex to highlight the concept's generality

Third, let's *define a desirable experimental design for each coexistence mechanism*, *using common terms*. In Figure 1, I propose a research landscape for experimental designs, which corresponds to the hierarchical organization that is presented in Table 1. Given the consensus opinion about the importance of species density and frequency manipulations in coexistence experiments (Adler et al., 2018; Godoy et al., 2017; Hart et al., 2019; Luimstra et al., 2020; Wainwright et al., 2019), the core design that should be included in any coexistence experiment is the assessment of the target species’ population response when each species is maintained alone and in a species mixture, at low and high densities (Figure 1's center). Note that despite this consensus, today, many coexistence experiments have solely focused on a partial set of these density and frequency components. For example, experiments testing the network‐dependent mechanism, the natural enemy partitioning mechanism, and the storage effect, often, do not include treatments that mix the species at different frequencies, while experiments testing the resource‐ratio and FD‐exploitation mechanisms rarely manipulate the total species densities (see papers reviewed by Comita et al., 2014; Kuang & Chesson, 2010; Levine et al., 2017; Sherratt & Harvey, 1993; Wilson et al., 2007). The elements at the landscape's edges represent the additional components that are required for testing specific mechanisms. These elements depend on the phenomenological approach (left and right sides of the figure) and the reliance of the interactions between species on environmental fluctuations (upper and lower parts of the figure's right side), species traits (right corner at the bottom), and extra biotic interactions (lower middle section). This landscape also reflects the similarity in experimental designs, where mechanisms that are either grouped into the same class (Table 1) or into adjacent classes that are divided by a dashed line can be simultaneously tested by the same experimental design. For example, each of the following pairs—(i) natural enemy partitioning and FF exploitation and (ii) the relative nonlinearity of competition and the storage effect mechanisms—belong to a single class (Table 1) and are, thus, adjacent in the research landscape and can be explored by the same experimental design (Letten et al., 2018). Similarly, the mechanisms belonging to the fluctuation‐dependent class, and those belonging to the fluctuation‐independent class, which are situated close to each other in the research landscape, can also be explored simultaneously by an extended experimental design. In such a design, the environmental conditions that manifest trait differences between species or the presence of extra biotic interactions under various environmental conditions may be manipulated (Kuang & Chesson, 2010; Figure 1).

Finally, let's *formulate key long*‐*term*, *research questions for assessing the application of the coexistence theory to natural communities*. This will set priorities in coexistence research. For example, if a key question addressed the association between a group of organisms and a specific coexistence mechanism, a high priority would be to extend the experimental scope to more model organisms and to adjust existing simulation‐based approaches (Ellner et al., 2019) to a wider range of organisms.

Using common terms and operational predictions, following a uniform research landscape of experimental designs will identify coexisting species and their underlying coexistence mechanisms in disease and other understudied systems, moving coexistence research forward!

## AUTHOR CONTRIBUTION

**Hadas Hawlena:** Conceptualization (lead); Funding acquisition (lead); Investigation (lead); Project administration (lead); Writing – original draft (lead); Writing – review & editing (lead).

## CONFLICT OF INTEREST

The author declares no competing interests.

## Data Availability

Data sharing is not applicable to this article as no new data were created or analyzed in this study.
